# Renal Carcinoma and Kartagener Syndrome: An Unusual Association

**DOI:** 10.1155/2020/8260191

**Published:** 2020-05-16

**Authors:** Hamza Dergamoun, Abdelilah El Alaoui, Imad Boualaoui, Hachem Sayegh, Lounis Benslimane, Yassine Nouini

**Affiliations:** Department of Urology A, Ibn Sina Hospital, Mohammed V University, Rabat, Morocco

## Abstract

**Background:**

The association of renal cell carcinoma and Kartagener's syndrome is unusual, and only eleven cases have been reported in the literature. The purpose of this work is to analyze this unusual association of Kartagener's syndrome and renal cell tumor and to study the main diagnostic and therapeutic aspects through our observation and review of the literature. *Case Presentation*. We report the case of a 50-year-old patient, with a history of recurrent respiratory infections, in whom a renal tumor was simultaneously diagnosed with Kartagener's syndrome, represented by situs inversus, bronchiectasia, and chronic sinusitis. The patient was treated by partial nephrectomy, and the histological examination showed a clear cell carcinoma. Through this observation and a review of the literature, we try to analyze this association as well as the main diagnostic and therapeutic aspects.

**Conclusion:**

The association of situs inversus and renal cell carcinoma is very rare—preoperative assessment and anatomy knowledge are crucial for a better adaptation of the surgical technique.

## 1. Background

Kartagener's syndrome is rare. Bronchiectasis, chronic sinusitis, and situs inversus are the classic three abnormalities. This syndrome is a part of primary ciliary dyskinesias (PCD), a rare genetic group of diseases that involve a constitutional abnormality of the cilia leading to respiratory damages [1, 2]. More rarely, PCD may be associated with cardiac, renal, or sensory defects, resulting in complex phenotypes. There is no known evidence that situs inversus increases the risk of malignancy [3]. The association of renal cell carcinoma and Kartagener's syndrome is unusual, and only 11 cases have been reported in the literature.

We report a case of renal cell carcinoma (RCC) discovered in a patient with Kartagener's syndrome. According to our knowledge, this case is the first reported in our country, and the 2^nd^ case in the world treated with partial nephrectomy.

Through this observation and a review of the literature, we try to analyze this association as well as the main diagnostic and therapeutic aspects.

## 2. Case Presentation

We report the case of 50-year-old patient, married for 12 years, without children, a regular chronic smoker (30 pack years), who suffered from recurrent pulmonary infections. He consulted for right lumbago beginning 8 months earlier, nonrelieved by analgesic treatment. The patient's overall condition was preserved and no hematuria has been noticed.

**Figure 1:**
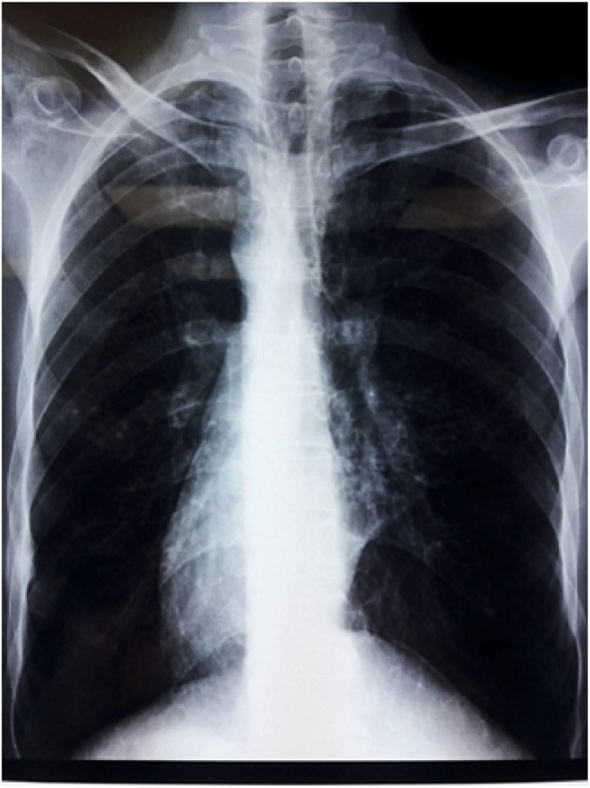
Chest X-ray showing dextrocardia.

An abdominal ultrasound showed cystic lesions of the right kidney containing multiple thick septa, without vegetation, measured 73 × 72 mm. The liver is found in the left hypochondriac region and the spleen on the right one, confirming the situs inversus. A chest X-ray revealed dextrocardia (Figure 1).

**Figure 2:**
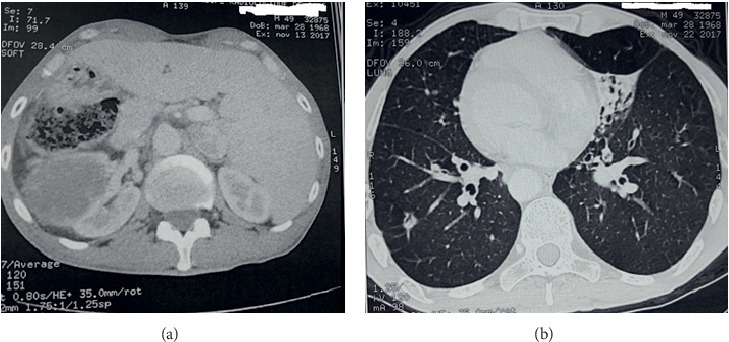
Computed tomography demonstrated a mass in the right kidney and mirror-image organs with left-to-right reversal (a) and bronchiectasia with situs inversus (b).

Enhanced abdominal computed tomography (CT) has confirmed the situs inversus and showed a bulky mass in the right kidney, measuring 7 cm × 8 cm (anterolateral and craniocaudal) with cystic patches and multiple images of thickened septa (Figure 2). There were no tumor thrombus in the renal vein or the inferior vena cava.

Functional respiratory tests were also performed and revealed an irreversible obstructive airway disorder and pulmonary hyperinflation. Azoospermia was found in the sperm test, preoperative renal function was normal, and the rest of biological assessment showed no other abnormalities.

**Figure 3:**
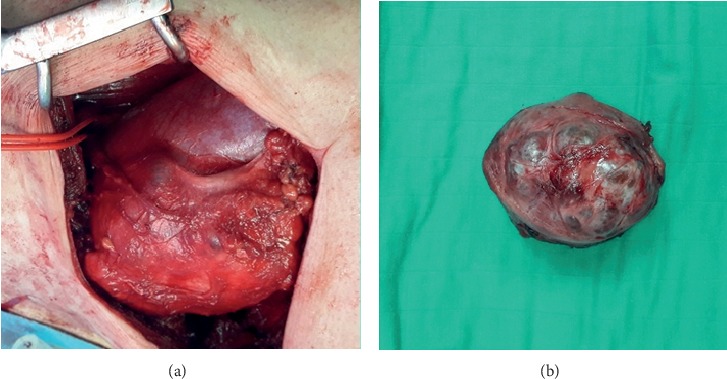
Intraoperative image showing the kidney and the tumor lesion (a) and specimen of partial nephrectomy (b).

We diagnosed our patient as having a T2bN0M0 renal tumor. The favorable RENAL nephrometry score (6a) allow us to put forward a partial nephrectomy with open approach, by a flank incision along the eleventh intercostal space (Figure 3). The histologic examination of the surgical specimen revealed a renal cell carcinoma—the margins were not affected.

In immediate postoperative, the patient has presented with a hematuria but the renal function was still be normal. The patient was discharged at postoperative on day 5. *In the absence of standardized data*, we plan to carry out a follow-up according to the recommendations of the *French Association of Urology* of a renal tumor with low risk, either the realization of a scanner at 6 months followed by every year for 3 years, a final control at 5 years and a monitoring of the renal function by assaying plasma creatinine at the same time as the scanner [4].

One year later, the patient is still alive, without any metastases and with a normal renal function.

## 3. Discussion

A review of the literature unveils the rarity of this association. Only eleven cases have been published. Bertini and Boileau described firstly the same case in a 54-year-old woman [5]. Oake and Drachenberg studied the operative considerations for the treatment of renal carcinoma in a 65-year-old man with situs inversus [6]. The age of patients varies between 43 and 83 years with only one woman and ten men. Six patients had the tumor in the left side when five in the right one. Nine patients had a curative treatment by radical nephrectomy, while only one palliative treatment and another treated by partial nephrectomy. Five cases of clear cell carcinoma had been reported (Table 1).

Surgery in these patients with situs inversus is particular. The modifications in surgical anatomy for the reversed abdominal organs complicates the surgical attitudes. Surgical treatment presents many technical issues; moreover, several surgical techniques have been described including laparoscopic techniques. To reduce these surgical difficulties, Makiyama et al. described the first case of a laparoscopic nephroureterectomy in a patient with situs inversus; the team developed a laparoscopic simulator specially dedicated to the reversed anatomy to allow preoperative training [7].

Patient's preoperative evaluation with CT scan and magnetic resonance imaging can be helpful to explore anatomical variations and determine surgical approach. Literature reports increased the frequency of vascular anomalies associated to the situs inversus; in addition to the transposed position of the aorta and vena cava, Makiyama et al. [7] in their case report found 2 renal arteries to the right kidney and a right renal vein passing over the aorta and ramifying an adrenal vein, a lumbar vein, and a gonadal vein. Oake and Drachenberg [6] report a case of patient with two renal veins and a number of extrarenal arteries travelling both anterior and posterior to the inferior vena cava.

The second case of renal cell carcinoma reported in a patient with situs inversus in the world mentioned a lower than normal origin of the left renal vein [8].

The management of renal vessels should be carefully performed because of the transposed position of the aorta and the inferior vena cava and the elevated incidence of associated vascular abnormalities.

Radiological image, such as 3D-constructed CT, may specify the different variations and facilitate the management of the surgical procedure. The use of magnetic resonance scanning preoperatively allows detailed planning of the approach as required.

The surgical approach is not standardized, and an exhaustive preoperative assessment (CT, MRS) seems to be the best way to orient towards the safest technique.

We did not use the laparoscopic approach in our case because of the rarity of that and lack of facility due to anatomical variations. We suppose that partial nephrectomy will be very difficult in laparoscopic approach.

Preoperative evaluation and postoperative follow-up are very important in these patients that have cardiac, pulmonary, and renal anomalies [8].

## 4. Conclusion

The association of situs inversus and renal cell carcinoma is very rare. To our knowledge, this is the first reported case in Morocco. Preoperative assessment and knowledge of anatomy are crucial and allow for better adaptation of the surgical technique.

## Figures and Tables

**Table 1:** Literature review: clinical cases published of situs inversus and renal carcinoma.

No.	Case report	Age/sex	Location	Open/Lap nephrectomy	Radical/partial nephrectomy	Histology
1	Bertini and Boileau 1987 [5]	54/F	Right	Open	Radical	_
2	Treiger et al. 1993 [8]	82/M	Left	Open	Radical	_
3	Adler and Lerner 1998 [3]	58/M	Left	Open	Radical	_
4	Jimenez Verdejo et al. 2000 [9]	43/M	Right	Open	Radical	_
5	Jewell and Bowa 2001 [10]	43/M	Left	Open	Radical	Clear cell
6	Takagi et al. 2008 [11]	50/M	Left	Open	Radical	_
7	Makiyama et al. 2012 [7]	61/M	Right	Lap	Radical	_
8	Rangarajan et al. 2013 [12]	65/M	Right	Nonsurgical	_	Clear cell
9	Terakawa et al. 2014 [13]	81/M	Left	Lap	Radical	Clear cell
10	Sağlam et al. 2015 [14]	52/M	Right	Open	Partial	Clear cell
11	Oake and Drachenberg 2017 [6]	65/M	Left	Open	Radical	Clear cell
12	Our study	50/M	Right	Open	Partial	Clear cell

F: female; Lap: laparoscopic; M: male.

## Abbreviations

PCD: Primary ciliary dyskinesias
VHL: von Hippel-Lindau disease
CT: Computed tomography
RCC: Renal cell carcinoma
MRS: Magnetic resonance scanning.
